# Electricity: Its Use in Hysteria

**Published:** 1869-01-15

**Authors:** James T. Newman

**Affiliations:** Chicago


### *Electricity: Its Use in Hysteria.*

BY JAMES T. NEWMAN, M. D., CHICAGO.

Was called upon the 1st inst., at half-past nine in the evening, to see a young woman who was suffering with hysteria. The paroxysm had been brought on by excessive grief. Ordered her to have a warm foot bath, after which she was put in bed. It was hardly of any use, because two strong men could with difficulty control her raving, yet she was small and very frail. The fits would come on every five or ten minutes, and last equally as long. Prescribed the following:

℞. Assafœtidae ʒj; (rub well, add)

Aqua Mentha pip. ℥ vi;

Tinct. Valerianac Ammoniæ ʒ ii;

" Castorei ʒ iii;

Ætheris Sulphurici ʒj;

M. Sig. A teaspoonful hourly during the night.

Visited her in the morning; found her no better than when seen last, the only difference was that the spasms were longer going on and coming off. Ordered an enema of Assafœtida with mucilage acaci[illegible]. Prescribed at the same time:

R. Zinci Valerianatis, gr. xii;
Potassii Bromidi, gr. xx;
Misce fiant in chart. iv;
Sig. One every four hours.

January the 2d, at four o'clock in the evening, saw her again; found her frothing at the mouth, and evidently worse. She had taken a small quantity of soup at noon, the only nourishment received since taken. I knew she could not long withstand such powerful straining and jerking of the limbs, and my faith was so strong in the nervous sedatives, that I resolved on repeating the powders, with the exception of adding morphiæ and changing quantities, as follows:

℞. Zinci Valerianatis, gr. xx;
Potassii Bromid, gr. xii;
Morphiæ Sulphatis, gr. iv;
Misce fiant in chart., No. vi;
Sig. One every two hours.

She went to sleep at midnight and rested quietly all night, when all at once the paroxysms broke forth with redoubled fury; the rest she obtained seemed only to increase their power. Ordered her to have some beef tea instead of water when she wanted to drink. There she lay raving and tossing like a maniac; medicine seemed of no use. I determined to change my course of treatment. I thought of electricity, and acted on the impression. Took the electro-magnetic battery and placed one of the poles to the occiput and the other to sacrum; the effect was instantaneous, the rigidity of the muscles gave way. She soon went to sleep, and had no more paroxysms that day. In the morning, January 4th, I made another application, and to use her own words, she said it gave her strength and cheerfulness. She recovered rapidly, and soon went about her work as usual.

Case 2d, Jan. 9th, 1869.—Was called to see a married woman aged 36 years. The poor woman had suffered both mentally and physically at the hands of her cruel husband, and to cap the climax of his meanness he had sold all the little treasures given her by her mother. Pardon me for taking up so much space, I will now come to the case.

Found her laboring under an attack of hysteria, frothing at the mouth, twitching and jerking of the limbs. I resolved to give the electro-magnetic battery another trial, which I did, and to my great pleasure the effect was magical. She has not had any more paroxysms since I first used the battery. By the way, I neglected to say that she had the spells about a week before I was called. I have just left her house, and she says that she feels better than she has felt for a long time. In recording this case I have come to the conclusion, that much good may result from the use of electricity as a therapeutic agent, but we must not expect too much from it, for the simple reason that we have no specifics.

